# Integration of Distributed Services and Hybrid Models Based on Process Choreography to Predict and Detect Type 2 Diabetes

**DOI:** 10.3390/s18010079

**Published:** 2017-12-29

**Authors:** Antonio Martinez-Millana, Jose-Luis Bayo-Monton, María Argente-Pla, Carlos Fernandez-Llatas, Juan Francisco Merino-Torres, Vicente Traver-Salcedo

**Affiliations:** 1 Instituto Universitario de Investigación de Aplicaciones de las Tecnologías de la Información y de las Comunicaciones Avanzadas (ITACA), Universitat Politecnica de Valencia, Camino de Vera S/N, Valencia 46022, Spain; jobamon@itaca.upv.es (J.-L.B.-M.); carferll@itaca.upv.es (C.F.-L.); vtraver@itaca.upv.es (V.T.-S.); 2 Servicio de Endocrinología y Nutrición del Hospital Universitario y Politécnico La Fe, Bulevar Sur S/N, Valencia 46026, Spain; mariaargentepla@gmail.com (M.A.-P.); merino_jfr@gva.es (J.F.M.-T.); 3 Unidad Mixta de Investigación de Endocrinología, Nutrición y Dietética, Instituto de Investigación Sanitaria del Hospital Universitario y Politécnico La Fe, Bulevar Sur S/N, Valencia 46026, Spain; 4 Unidad Mixta de Reingeniería de Procesos Sociosanitarios (eRPSS), Instituto de Investigación Sanitaria del Hospital Universitario y Politecnico La Fe, Bulevar Sur S/N, Valencia 46026, Spain

**Keywords:** type 2 diabetes, risk models, service-oriented architecture, system integration, system reliability pilot, decision making, health care

## Abstract

Life expectancy is increasing and, so, the years that patients have to live with chronic diseases and co-morbidities. Type 2 diabetes is one of the most prevalent chronic diseases, specifically linked to being overweight and ages over sixty. Recent studies have demonstrated the effectiveness of new strategies to delay and even prevent the onset of type 2 diabetes by a combination of active and healthy lifestyle on cohorts of mid to high risk subjects. Prospective research has been driven on large groups of the population to build risk scores that aim to obtain a rule for the classification of patients according to the odds for developing the disease. Currently, there are more than two hundred models and risk scores for doing this, but a few have been properly evaluated in external groups and integrated into a clinical application for decision support. In this paper, we present a novel system architecture based on service choreography and hybrid modeling, which enables a distributed integration of clinical databases, statistical and mathematical engines and web interfaces to be deployed in a clinical setting. The system was assessed during an eight-week continuous period with eight endocrinologists of a hospital who evaluated up to 8080 patients with seven different type 2 diabetes risk models implemented in two mathematical engines. Throughput was assessed as a matter of technical key performance indicators, confirming the reliability and efficiency of the proposed architecture to integrate hybrid artificial intelligence tools into daily clinical routine to identify high risk subjects.

## 1. Introduction

Diabetes is a set of pathological disorders related to an impaired insulin production and/or action [1]. Specifically, Type 2 Diabetes Mellitus (T2DM) is characterized by both an insulin action resistance and a progressive dysfunction of the endogenous insulin release process. It differs from other types of diabetes by the triggering factor, which is related to unhealthy lifestyle and the long-term defect originated by aging [2]. T2DM prevalence is rapidly rising throughout all the world [3]. In 2013, there were 382 million people with T2DM, and there are estimates of the proportion of undiagnosed diabetes accounting for 30% of the population [4].

The diagnostic test to confirm T2DM is based on the comparison of laboratory tests and specific ranges [5]. Even though the fasting glucose and the HbA1C are used to identify subjects at high risk of acquiring T2DM, the gold standard test is the Oral Glucose Tolerance test at 2 h (2h-OGTT). In this test, the subject intakes a 75-g dose of glucose diluted in 3 dL of water (concentration <25 g/dL) through the oral way in less than 5 min. Prior to the test, the subject has to achieve a basal metabolic performance by a specific food prescription, glucose-related drugs abstention and fasting for 8 h.

Clinical researchers and epidemiologists are striving to produce classification algorithms and predictive models to understand why individuals develop this type of diabetes [6,7]. The benefits of the early detection of pre-diabetic stages are extensively confirmed by the literature [8]. In this context, the use of modeling techniques has become popular with a wide range of research-based tools to detect individuals with a high risk of developing T2DM [9]. Although in the European countries, screening questionnaires continue to be extensively used to collect source data, there is a continuously growing set of electronic health records in both secondary and primary care, which could be used to develop and validate predictive algorithms [10].

A T2DM risk score has to accurately estimate the risk of a subject to develop T2DM [11]. This scoring can be either based on a numerical discrimination, which assigns an individual a numeric value, or on a qualitative risk prediction, on the basis of high, mid and low probability of developing T2DM in the future. Discrimination and prediction algorithms are statistical models that combine information from several sources of clinical and lifestyle data. Common types of models include logistic regression models, Bayesian networks, support vector machines, Cox proportional hazards models and classification trees [12], and each type of model produces an individual risk based on the individual data. However, various factors can lead a risk score to perform poorly when applied to other individuals, and even to other populations [9]. It may happen that a model prediction is not reproducible because of deficiencies in the baseline data (missing values, erroneous data) or modeling methods used in the study in which the model is derived, mostly due to over-fitting, differences between patient characteristics, measurement methods, health care systems particularities or data quality [13].

Risk score validation requires a full specification of the existing model (that is, both the input variables and their weights) to predict the outcome. Such specification should also include the development strategy (training and validation), and if applicable, the comparison of the model predictions and the real patient outcomes (discrimination analysis). Few predictive models are used in clinical practice, most probably because of a lack of external validation [14,15]. Moreover, the majority of the models published in the literature require the collection of data that are not available in the healthcare system, as they are obtained under the execution of a clinical trial [9,12].

A risk score should be clinically credible, accurate (well calibrated with good discriminative ability), have generality (be externally validated) and, ideally, be shown to be clinically effective; that is, provide useful additional information to professionals that improves decision making and thus patient outcome [15]. It is crucial to quantify the performance and importance of a predictive model on a new series of patients before applying the model in daily practice to guide patient care [16]. There are several criteria for assessing the selection of a decision support tool, but it should include the widely-known indicators of effectiveness (sensitivity and predictive value), the predictive power and application to all risk categories [17]. Moreover, its accessibility to the clinical staff, the possibility for time-line evaluations (provide a baseline to evaluate the intervention over time or costs), the ease of use and positioning to support wider considerations should also be considered.

The combination of different modeling techniques may be a solution towards under-performing risk scores [18]. Hybrid modeling consists of mixing different modeling approaches over a high-dimensional set of data to maximize the discrimination likelihood [19], which is extensively used for research purposes and to produce T2DM risk scores [20,21]. However, the real implementation of such mixed models remains challenging in clinical settings, as many confluent factors related to the technological framework, access to mathematical engines and data quality hinder their application to identify high risk patients in a reliable way.

To this end, we propose a distributed heterogeneous architectures as a solution to meet the needs reported above. The specification of a model is usually approached by mathematicians and bio-statisticians; afterwards, the model is wrapped into software pieces by designers and computer engineers and finally used by clinicians in a web or desktop application. The interaction of these stakeholders during the design, development and release of the decision support tool for the pre-diabetic screening is a process that has to be coordinated and well documented. In this paper, a novel architecture to overcome the main limitations of the validation of discrimination and prediction models is presented. Our principal aim is to provide a platform capable of translating clinical research on T2DM risk scores to a real setting and to promote evidence-based medicine. Our approach is to use a common data repository structure integrating several real data sources from the Hospital Information System (HIS) and to build upon a system in which independent components can be executed according to a predefined workflow and be used by endocrinologists.

This work describes in detail a working release of a decision support system comprised of a distributed architecture, with an associated ontology, mathematical modeling algorithms and the protocols for clinical information exchange. The system was tested in a clinical pilot to assess the feasibility, reliability and effectiveness of integrating risk scores in clinical facilities by monitoring technical indicators.

Our results confirm that the approach is adequate to integrate complex modeling techniques for clinical case revision on daily basis. Security and privacy issues are granted with the use of a distributed data warehouse. The scalability and reliability of the model execution over large datasets is also granted by distributing technologies.

The manuscript is structured as follows. First, a background of the techniques for data modeling on T2DM risk scores, the data infrastructure needs and the business context are presented. Afterwards, the architectural specification and the description of the implementation are presented, also showing the results of the three-month clinical trial. The manuscript concludes by reviewing the achieved results and providing guidelines for future work.

## 2. Materials and Methods

The main purpose of this research is to provide a technological structure in which the clinical research can be straightforwardly applied to patients and then make decisions based on medical evidence.

Our approach is to define, implement and assess a distributed architecture capable of integrating hybrid modeling to discriminate patients at high risk of developing T2DM. Sackett defines the practice of evidence-based medicine as a life-long, self-directed learning process in which caring for patients creates the need for clinically-relevant information about diagnosis, prognosis and therapy [22]. Such a paradigm has to: (1) convert data into answerable questions; (2) track down the best evidence to answer them; (3) critically appraise that evidence for its validity (closeness to the truth) and usefulness (clinical applicability); (4) integrate this appraisal with our clinical expertise and apply it in practice; and (5) evaluate its performance. Embracing this definition, we first had to define the business context, with a proper identification of the stakeholders and their environment.

### 2.1. Business Context Definition

The business context of the system to support the execution of T2DM risk models in clinical settings is based on the stakeholders and the offered services (functionalities).

Stakeholders are the abstract roles who use the system from different perspectives and for different purposes (viewpoints). Considered stakeholders and their own viewpoints are:

- End users: non-technical end users such as health care professionals, health care managers, patients and citizens; health professionals, including managers and policy makers and medical researchers who are concerned with public health affairs. Good development environments and friendly interfaces will lead to better quality software and will attract professionals to use the tools. Efficient communication among service providers would result in services that better meet end user requirements.
- Service providers are concerned about the commercial exploitation of the system. They need to maintain an effective communication with their end users and a fluent interaction with the runtime environment to explore potential integrations.
- Researchers are mainly concerned with good development environments, a knowledgeable community of developers and access to resources for implementing software and algorithms. The system should support researchers as a major stakeholder and allow them to participate in the system improvement (together with service providers and end users). Two main domains of research are found within this viewpoint: data mining research and software research. The first type is focused on the development of new algorithms and models to perform stratification and variable association analysis. The second type aims to improve the software quality of the services, interfaces and database management.

These requirements can by turned into functionalities and classified under modules, which are the entities that provide services and operate within the system. These modules may offer services to be consumed among themselves or directly by stakeholders:

- The Data Storage module is in charge of providing a warehouse for all the data within the system. From a conceptual point of view, the data model is unique for all of the system, containing Electronic Health Records (EHRs) and other kinds of data (logistic and administrative).
- The Model Host module is the core of the system. It is in charge of managing the client requests (user interactions), running the risk scores and querying the data warehouses. It gathers into an application server the tools (models) that will run the algorithms over data from EHRs and provides the services for managing them from the client side. The Model Host module will also contain components to provide horizontal services including security features, tracking and system management.
- The Plug-in module is the part of the system that hosts the user interfaces. These user interfaces are web pages formatted for the intended use for each type of user and scenario. The integration with existing disease management systems is articulated wrapping the interfaces within plug-ins, tailored for each integration case.

**Figure 1:**
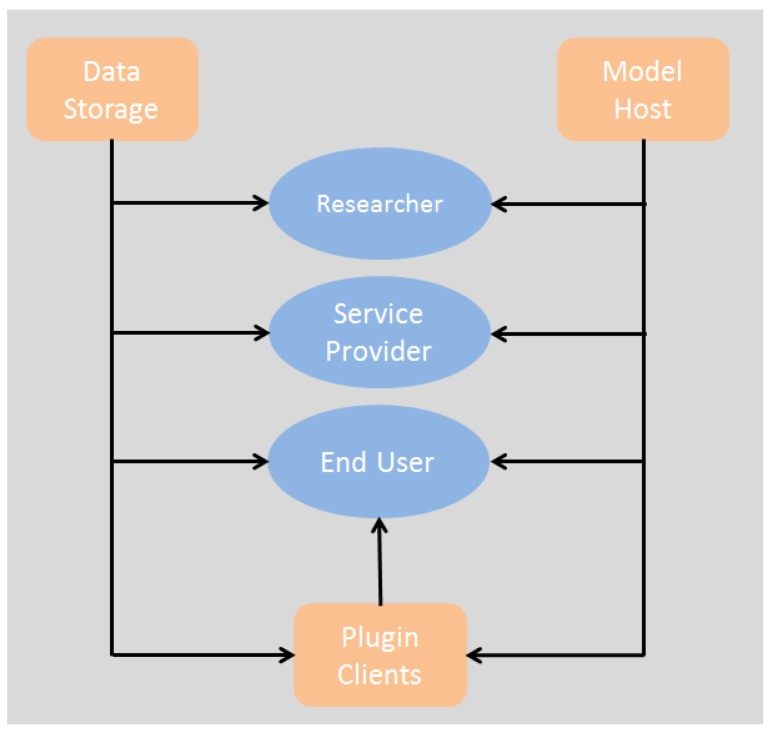
Business context showing the relationships among services and stakeholders.

The list of stakeholders above is highly generalized; however, it provides a good division of the roles and services that build up the system architecture. Figure 1 maps each stakeholder category to each of the conceptual models of the proposed architecture. In our approach, only the end users have a relationship with the three modules (plugin, data storage, model host), whereas researchers and service providers are only related to the model host and the data storage. Moreover, this figure shows that the plugin module is dependent on the characteristics of the model host and the data storage; however, these two latter components are independent (have no arrows between them).

After having identified relevant stakeholders, we had to look into and understand their expectations, i.e., the expected benefits the system would provide them, and define the reference quality metrics to satisfy their expectations.

### 2.2. Quality Metrics

The defined business context should provide a mapping among use cases that evidence stakeholders’ expectations in terms of reference services. The ISO/IEEE 1471 methodology has been used to perform the mapping between the system architecture and the stakeholders’ expectations. The requirements represented by the study scenarios (and their technical specifications) provide a set of measurable constraints on the architecture to measure its conformance. Emerging from the stakeholders’ perspectives and the scenarios, three categories have been defined:

- **Category 1**: a system for running algorithms on demand with a specific running environment regardless of patient health records or additional data than a set of defined parameters.
- **Category 2**: a system for running algorithms on demand with a specific running environment, which needs patient health records and additional data form a huge amount of variable parameters.
- **Category 3**: a system for running algorithms on demand at the client side with a specific running environment, which needs raw and pre-processed data.

Extracted from these three categories can be identified a set of common pathways. Based on ISO/IEEE 1471 [23], a second level of abstraction is needed to draw the common concepts or processes within these tools. Moreover, ISO 18308 describes the reference methodology for describing a software architecture and also for identifying the requirements for a successful electronic health record system integration [24]. These two standards were used to define the reference success criteria indicators, depicted in Table 1.

### 2.3. Business Environment Definition

The business environment is defined by mapping the business context into real deployable components. UML is a markup language that allows one to perform this mapping by defining the structural aspects of the components. System modules for the Data Storage, Model Host and Plug- in of Figure 1 are mapped into high-level components that will implement the services (low-level definition). Users of the system (researchers and end users) will define the characteristics of the components, and this definition will be used for the description of the services in the system architecture. It is important to highlight that although there are services rendered by the system modules, in further applications (concrete architectures), each service could be provided by a separate business entity, deployed and operated independently, with the only requirement being compliant with the inter-operable service protocol.

**Figure 2:**
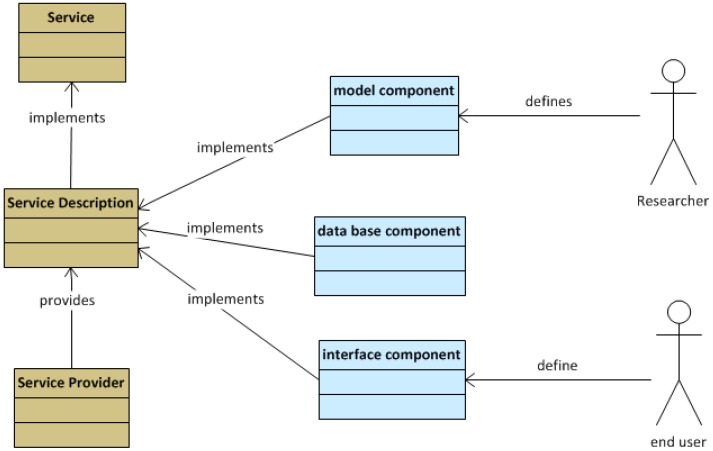
UML component and service descriptors.

Major information concepts that are used to qualify the provided services are described by means of UML descriptors (Figure 2). These concepts are mainly related to the offered services and how the architecture handles and processes those services in general, helping to contextualize their use.

The information regarding each service is stored in the platform in the form of a service description using the Web Service Description Language (WSDL). As proposed by [25], this description contains references to the implementation of the service on an XML basis. In the system architecture, a service is constituted by one or many components that belong to a specific system module. A service might also be constituted by other services (a composed service), and in this case, the service description will have a reference to the other services’ descriptions.

### 2.4. Service Collaboration Pattern

In the following section, we will look into the details of each of the three system modules and their components. The goal is to identify the high-level reference services that are provided at two different levels, as shown in Table 2.

The system architecture has been designed as a Service-Oriented Architecture (SOA) [26], in which the different components from different modules access the whole functionality of the system that may be located in different physical allocations (one or several servers) through a set of web services. These components interact with each other over the Internet in a modality prescribed by its description using SOAP messages, conveyed using HTTPS with an XML serialization in conjunction with other web-related standards.

Services are listed depending on their nature and purposes; for this reason, they have been gathered in several different components, which pertain to each of the three modules.

### 2.5. Data Warehouse Infrastructure

A data warehouse is composed of one or more databases or subsets of data, also known as data marts, which store heterogeneous data models and structures. This heterogeneity makes it difficult to develop efficient querying functions for data warehouses [27,28]. Use of knowledge domain descriptors and semantic references, through the definition of an ontology, is key to formalize and map the type of data hosted in a data warehouse [29].

Even though classic SQL engines are still hard to beat (Table 3), there are several commercial and non-commercial database engines with top featured options on volume, variety, speed and reliability such as the MongoDB and NoSQL systems. However, regardless of the engine performance, interoperability is a key factor to design a proper Data Warehousing (DW) system.

To this end, Informatics for Integrating Biology and the Bedside (I2B2) is one of seven centers funded by the NIH Roadmap for Biomedical Computing. The mission of I2B2 is to provide clinical investigators with a software infrastructure able to integrate clinical records and research data.

The I2B2 architecture is made up of three layers: a Presentation Layer, a Service Layer and a Data Layer. The user accesses I2B2 at the Presentation Layer, which exposes a User Interface (UI) either through a web client or a local application.

Data are stored in the Data Layer, which contains the I2B2 DW. The only way the UI can access data is through the Service Layer. This layer is a collection of web services, each one denoted as a “cell”. The collection of these cells makes up the “I2B2 hive”. The main cells in the hive are: the Project Management (PM) cell, the Clinical Research Chart (CRC) cell and the Ontology Management (ONT) cell.

The PM cell accesses a set of data structures in the DW that associate users with passwords, preferences and projects. When a user logs on to the I2B2 web client, the PM cell manages the authentication process. Every time another part of the hive tries to perform an action on behalf of the user, it goes to the project management cell to gather the proper authorizations. Once authenticated, the user (through the web client) performs queries through the CRC cell, also known as the data repository cell. To facilitate the query process for the user, data are mapped to concepts organized in an ontology-like structure, which is managed and accessed by the ONT cell.

The I2B2 data model is based on a “star schema”. The star schema has a central “fact” table where each row represents a single fact. In I2B2, a fact is an observation about a patient. Observations about a patient are recorded by a specific observer in a specific time range (defined by start and end dates) and are related to a specific concept, such as a lab test or diagnosis, in the context of an encounter or visit. The concept can be any coded attribute about the patient, such as a code for a disease, a medication or a specific test result. This way of representing concepts is based on prior work known as the Entity-Attribute-Value (EAV) model [30]. The reason why the I2B2 developers decided to implement this model is that querying data modeled with a star schema represented in an EAV format is efficient [31].

### 2.6. T2DM Risk Scores

State of the art T2DM risk models are based on mathematical models executed on numerical and/or categorical variable (Table 4). Depending on the output, such models can provide the probability *p* of developing or having T2DM (Equations (1) and (3)) or the hazard rate of developing T2DM over time (Equation (2)). The performance of a model discrimination is assessed by the C statistic (also known as area under the curve of the receiver operating characteristics) [12].

(1) $$p=\frac{1}{1+\exp(-\left(\alpha +\beta_{1}X_{1}+\beta_{2}X_{2}+\ldots +\beta_{m}X_{m}\right))}$$

(2) $$h\left(t\right)=h_{0}\left(t\right)\exp\left(\beta_{1}X_{1}+\beta_{2}X_{2}+\ldots +\beta_{m}X_{m}\right)$$

(3) $$p=\alpha +\beta_{1}X_{1}+\beta_{2}X_{2}+\ldots +\beta_{m}X_{m}$$

where:

- α is the intercept or prior probability.
- $h_{0}\left(t\right)$ is the intercept baseline hazard rate.
- $\beta_{x}$ is the regression coefficient, which denotes the relative weight of the corresponding predictor.
- $X_{x}$ are the predictors or variables, which can be numerical (continuous) or categorical (0, 1, 2…).

One interesting model for T2DM detection, which is not based on the aforementioned regressions, is the MOSAIC model [40], which is open source and available for research (https://github.com/sambofra/bnstruct (last accessed 15 December 2017)). This model is based on a Bayesian network to impute unknown parameters. The MOSAIC model was built to be applicable in different contexts, and the performances are comparable to the Findrisc score in scenarios where clinical data are not available. This model shows an acceptable predictive value when clinical information is available for cholesterol and fasting glucose [41], so it was chosen as the missing data imputation methodology.

### 2.7. Design of the Pilot Study

The pilot study was based on a single center randomized study investigating the performance of the system and the scalability of the tools having real doctors using the tools. The evaluation consisted of nine consecutive weeks for assessing prediction and detection performance of T2DM risk scores on a real population, based on retrospective Electronic Health Records (EHRs). The biomedical research ethics committee of the Hospital La Fe approved in January 2015 the formal request of data and the study design. No further considerations were given by this committee.

The system was evaluated in the Endocrinology Service of Hospital La Fe during a continuous period of three months involving endocrinologists and the head of service, who used the tool for 2 h per session. Three training sessions were planned with the participants prior to the utilization. Participants who signed the informed consent were blindly randomized and assigned into the evaluation session schedule.The study plan consisted of three stages:

1. Training sessions: three group sessions for introducing participants to the tools and learning the actions to visualize data and execute the risk models.
2. Evaluation of risk scores and clinical evaluation: evaluation of the tools during sessions of 2 h during eight weeks.
3. Data analysis: acquisition of logs, traces and Key Performance Indicators (KPIs) for the technical assessment of the system.

For each model, a scenario for the best and worst case was defined according to the specifications and behavior of the operations. For the prediction model, the best case was the execution for a single patient, and the worst was the execution for the highest available population, which is 8080. In the case of the detection model, it can be executed only for a single patient, so the worst case is when the model did not have any input variable (i.e., it had to estimate the 21 missing parameters and the best case when it had 20 input parameters and only had to estimate one).

The technical throughput of the tool was assessed for the following KPI for the best and worst scenarios:

- Computational load (memory footprint on the server).
- Response delay to service request (s).
- Access time to main DB/cache (ms).
- Time usage span (s).
- Maximum response delay.

In order to confirm the scalability and reliability of the proposed architecture, it is of utmost importance to track the technical features. These two quality dimensions have been defined previously as the availability level and CPU-threshold-exceeded indicators [42,43]. To test these, we used the thresholds proposed by [44], which are CPU <83% and the availability level different from “unreachable”.

## 3. System Architecture Description

The system architecture is presented using the service-oriented architecture pattern, where services are provided and shared between the components within the three conceptual modules described in the business context. The communication is done using a communication protocol described in this section, which is controlled by a central component: the Choreographer. This section describes in detail the designed architecture. The first part of the description focuses on the type of services in the architecture. The second part describes the modules and the component. The third and final part describes the central component (Choreographer) and the communication protocol (XMGS).

### 3.1. Functional View

According to IEEE 42010 [45], the functional view describes the capabilities, structure, responsibilities and specifications of the system components and how they interact among each other. The functional view categorizes the services into three types: application, interoperability and system services.

**Figure 3:**
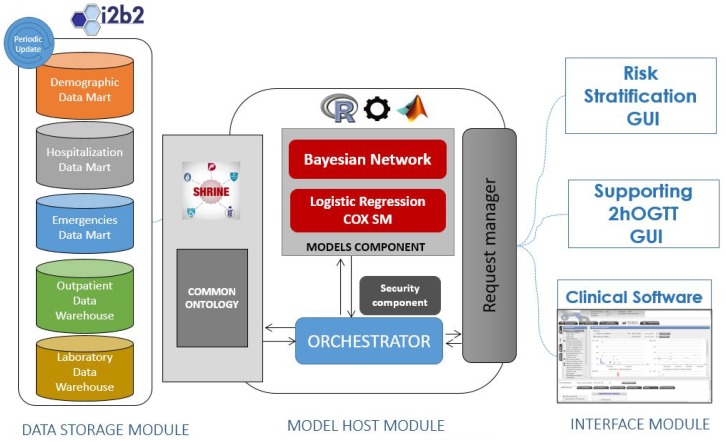
System architecture functional view.

Figure 3 depicts the system architecture and the functional relationships among the modules. The three modules are connected by the Choreographer. The services in the proposed architecture are grouped into three categories: Data Interoperability services, Modeling services and the User Interface services.

Data Interoperability services can be reused by any component within the system and are devoted to extract and store data from the Storage Module (e.g., can perform Extraction-Transform-Load processes that prepare input data for the algorithms or perform queries to display raw data in the interface). The Modeling services are services devoted to the execution of the prediction and detection algorithms. User Interface services cover the logic operations (including functional logic and infrastructure) that are common to multiple scenarios (for instance, showing data in the web interface or chart plotting).

From left to right in Figure 3, the schema shows the data storage module, based on the I2B2 technology, the Model Host module, which stores the hybrid models and the Choreographer, and last, the interface module containing the web applications through which the end users interact.

The Data Storage Module is composed of several single data entities from different sources (data marts): hospitalization, laboratory tests, outpatient services, etc. From a logical point of view, the Data Storage Module is a unique conceptual part, which is structured according to a common ontology presented previously [46]. This common ontology represents each clinical event happening to the patient at each data mart in the data warehouse, providing it with a start and end time and connecting it to the specific concepts related to a particular event. Once a query is prepared, the common ontology translates these concepts, and the Data Sharing Network (SHRINE) component aggregates the query to be executed on each of the data marts. From a physical point of view, each data mart is an isolated virtual machine located elsewhere and reachable though the Internet. The connection of the Data Storage Module and the Model Host Server is performed by the SHRINE service layer (delimited by a blue dotted line). SHRINE is composed by a set of interoperability services that allow performing federated queries to the whole data storage warehouses, regardless of its physical location and data structure [47]. This configuration allows researchers and clinicians to choose the target population to execute the models irrespective of the data source.

The rest of the services are gathered within the Choreographer component into the Model Host Module. The Choreographer is in charge of executing predefined work-flows for each GUI tool and model. As mentioned before, the requirements for providing the input parameters and running specific algorithms involve many software components within the system that must be able to work in a distributed and controlled way. This kind of complex process execution is solved by using the Choreographer, which assumes that the processes are able to exchange data to execute processes in a distributed way [48].

Service choreography allows services to communicate between them in what is known as the “defined execution flow” (workflow). Using this approach, it is possible to connect and disconnect components and modules dynamically. Components can provide and consume their services without the necessity of knowing the concrete architecture of the deployed service. This facilitates the creation of more independent and flexible services able to deal with different kinds of components and different configurations.

### 3.2. Model Host Component

**Figure 4:**
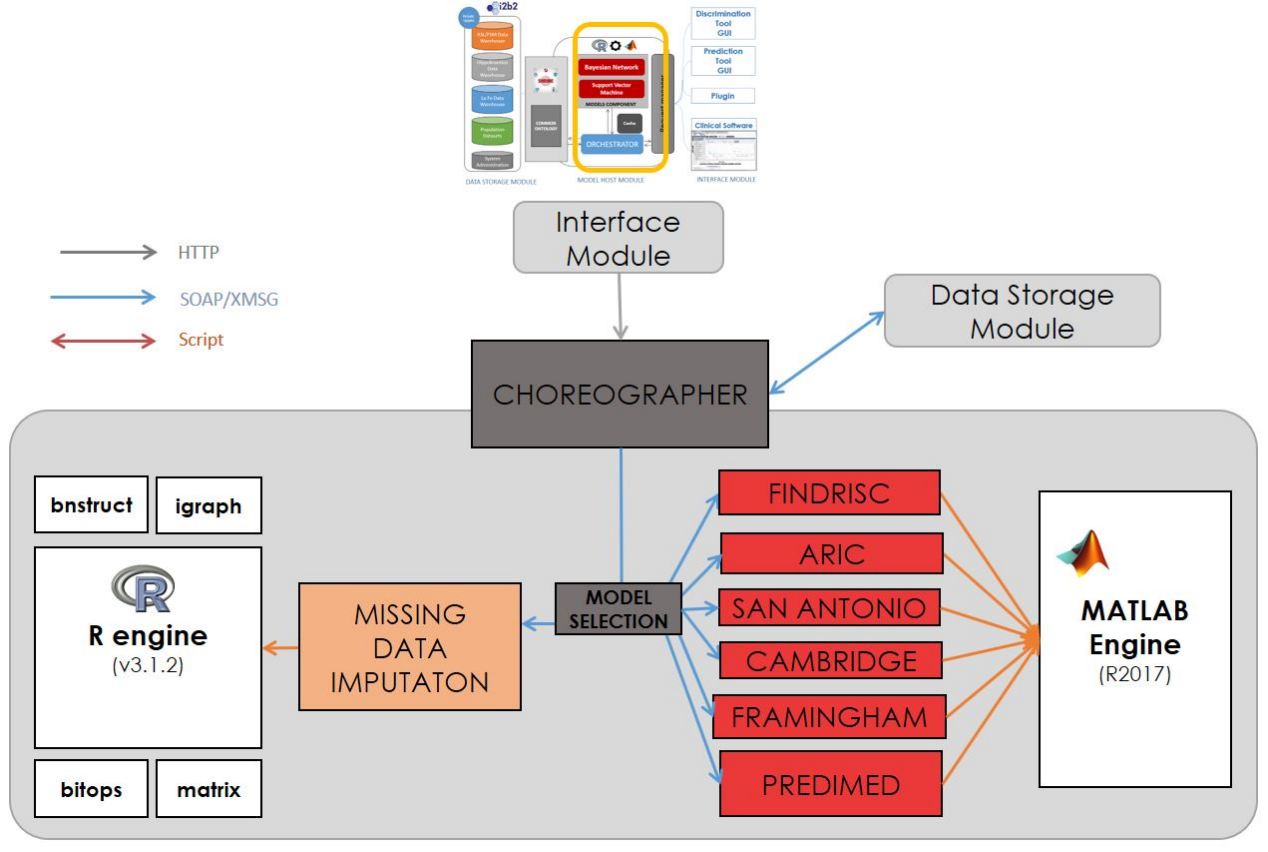
Execution of the risk score equations using mathematical engines.

Figure 4 shows the central part of the system architecture, which hosts the engines to execute risk scores. This section describes which services are provided from the components shown in the model host of Figure 3. As the components wrapping the models are continuously tested and refined, the services in this component are listed depending on the functionalities they provide (prediction or imputation), without listing the type and name of input/output parameters.

### 3.3. Security Component

The Security Componentis in charge of providing secure horizontal features for all the services and is part of the Choreographer. The security features are based on four dimensions:

- Authentication: It must be possible for the service provider to ascertain the identity of the service requester.
- Authorization: The service provider must be able to determine whether the requester has the appropriate rights to invoke the service.
- Message confidentiality: Message contents must only be visible to the intended recipient.
- Message integrity: It must be possible to guarantee that a message has not been altered or tampered with in transport between the service consumer and the service provider.

Authentication is supported through the use of client-side x.509 certificate, credentials (username and password) for each professional end user and a Security Assertion Markup Language (SAML) certificate. All web services are offered in a Secure Socket Layer (SSL), and the system implements this security feature encrypting the information exchanged between the end points; thus, the message confidentiality is guaranteed. Only certified connections will be accepted by this component. Each end user will be provided by a set of credentials (username and password), and it will be mandatory to log into the web applications and furthermore to authenticate the connection.

### 3.4. Track Component

**Figure 5:**
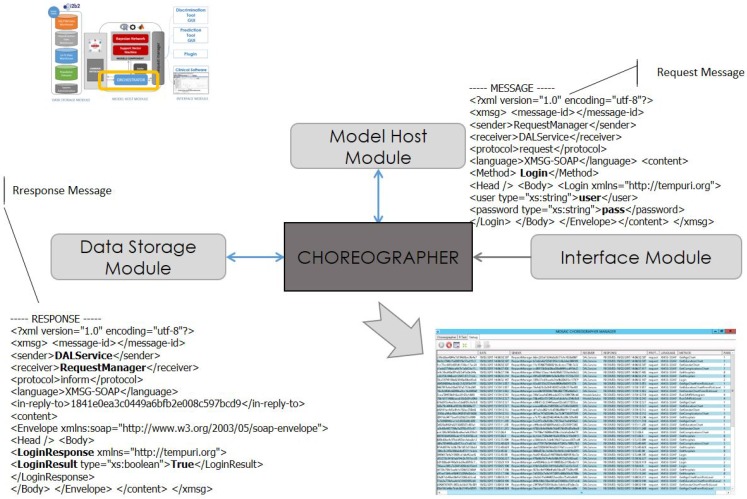
Tracking of the system service messages.

Every system must provide a record track of the executed services, their results, timestamps and other audit information. The track component is in charge of recording the trace of all the activities that take place during the performance of the system (in both test and deployment phases). The records must be standardized (or even normalized), understandable and be ready to be parsed and mined. Therefore, this component will record all the interaction events among the modules and components (Figure 5). As the user interaction deserves special attention and opens a brand new study field, all the interactions in the Interface Module will be recorded in a special format and placed in a basic txt file (to make access of the information easy). A file named LOGusername.txt will be automatically generated upon the first launch of a user. A main class controls the interaction events during a session and tracks them in that file. Each interaction event will be written in a line with the following format:

*<Time stamp>, <Module>, <control>, <Free text>*

- Time stamp: dd/mm/yyyy hh:mm:ss.
- Module: the module (view or form) in which the patient is currently.
- Control: the control used: button, label, picture, graph, chart, etc.
- Free text: free text that indicates the interaction or notes for the usability expert.

These messages are broadcast to one or more destinations and contain sections called appenders. There is a wide range of appenders; however, anyone can create their custom appenders, adding new information as the time stamp, running variables and extra information. Beyond the functionalities provided by third party libraries, such as Log4J, Log4Net and Google Log (Glog), the system offers two services to perform the program tracking and user interactions.

### 3.5. Communication Protocol

Following the SOA pattern, the choreography paradigm requires the use of a common interchange language that allows components to understand the purpose of the services available in the system architecture and information exchange.

Rather than using syntactical models with common message formats, the proposed approach aims to enhance the service descriptors using semantics. This is because the syntactical data format limits the capacity of services to understand the data content. This limitation can affect the independence of the services, which must be prepared to read data in all the possible formats and requires a strict subscription process to make sure that the component information is sent to the subscribed services. This is a problem in distributed architectures where the modules are exposed to a very aggressive and stressful environment in which the inner component configuration is continuously changing (e.g., a revision of a Bayesian network module to improve the model classification outcomes). The use of semantics as an alternative to syntactical models provides advantages for the overall system in the understanding of data structures and model execution.

**Figure 6:**
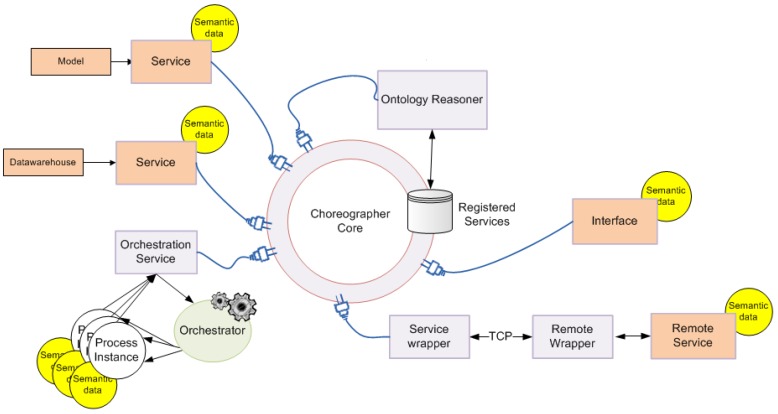
System Choreographer functional schema.

Figure 6 shows a picture of the system Choreographer. The core of the component is a message dispatcher engine (Choreographer) and a database that contains the services that are registered (declared) in the system. Services may be connected to the core locally, when the services are allocated in the same computer (e.g., Model Services), or remotely by using a TCP protocol service wrapper (e.g., SHIRNE services). An ontology reasoner is connected to the Choreographer, which is able to infer knowledge from registered services where semantic information is available through the core. Connected to the Choreographer is the Orchestrator service, which allows the use of workflows to execute predefined sets of actions.

Reasoners are software pieces that allow performing semantic search across the services. The semantical description of the services must provide a reliable shot of the functionalities and actions they provide so the core can detect automatically which services are available and what they intend to do. However, the key point of this component is the Orchestrator. It enables the execution of a predefined workflow, which describes sequential pattern of actions tailored for the automatic execution of processes. This component also allows one to produce graphical figures of the workflows to be interpreted by humans. The second component within the Models Host Module is the model grid, which contains the system services to run the algorithms (screening and risk classification) using the required running environment (R and MATLAB). Finally, the application services are located in the right part of the schema. They are the services that can be consumed by standalone tools and disease management systems that integrate these functionalities. In this study, the GUI tools were based on web applications (programmed in the bootstrap framework).

The Choreographer in the orchestrator component dispatches messages among the modules using a specific XML message protocol called XMSG. This protocol is based on the combination of the Foundation for Intelligent Physical Agents (FIPA) [49] and SOAP [26] protocols. The classic FIPA protocol, defined for multi-agent system communication, allows sharing knowledge using several protocols. XMSG is based on FIPA headers to route and characterize the messages. The content in XMSG is based in the SOAP protocol. SOAP is a well-known and widely-used protocol to perform service calls. The XMSG protocol allows broad and multi-cast, as well as P2P message calls using custom symbols in the destiny address.

An example of an XMSG message is shown in Table 5. The message is sent from the ModelService, to the LaunchRScriptmethod, whose logical address is *ModelService.R*. Both sender and receiver information and the type of message sent (request, inform, event, etc.) are defined in the message header. Following this, in the content part of the message, the call to the specific method of the service is defined. In this example, the method invoked is execute model, which needs the script and input parameters.

The communication among the services is done via peer to peer communications. Each service must know in each moment what services and methods are alive and what kind of information they are able to deliver.

## 4. Experimental Results

### 4.1. Scenarios for T2DM Risk Score Assessment

The expected impact of the system is to improve the characterization T2DM onset and target population at risk of developing T2DM in the future or which has already undiagnosed T2DM. Given as input the available variables in a electronic health record for a given patient or a given population, the models can estimate the probability of being at high risk and for detection models find out the most probable value of the diagnostic values [41].

Two different clinical scenarios (use cases) are defined into the screening and risk stratification:

1. Estimate missing variables given available variables measurable with a general practitioner’s visit and laboratory tests in the electronic health record towards risk stratification.
2. Estimate the 2h-Oral Glucose Tolerance Test (2h-OGTT) glucose range given all other available variables (supporting a diabetologist to decide whether this test is needed).

#### 4.1.1. Scenario 1: Risk Stratification

In this case, the input data are coming from the health information system of a healthcare institution or agency. The input data are demographics variables and, when available, some other variables measurable with a general practitioner’s visit and a blood test. The output will be a picture (through, say, a pie chart) of the distribution of the population most at risk of having T2DM and being pre-diabetic.

Case 1, healthcare agency with limited availability of EHRs: Let us suppose that the information available to the healthcare agency is limited to demographics variables (gender, age, etc.), because the health information system is still not integrated in these settings: before asking the hospital or the primary care institution to provide them with phenotype and metabolic information of their served population, this system could be used to better stratify this request and narrow it only to the population that actually has the highest probability of being at risk.

Case 2, healthcare agency with full availability of EHRs: In this case, the input data for the system will be all the variables usually available in a “normal” citizen’s clinical history record. The output will be used to determine the subgroups at risk of having T2DM or being pre-diabetic; another output could be the determination of other meta-variables like being a smoker, having high cholesterol or not having an optimal lifestyle. The tool could support decisions related to public health policies before conducting screening campaigns to better estimate their impact, e.g., how many 2h-OGTT tests are needed, fasting glucose blood tests, screening visits, etc.

Case 3, health insurance company: In this case, the system tool can be used to support the company in assessing the risk of healthcare expenses among a targeted group (a served company or group of individuals) and better develop routine activities such as finance forecasts, screening activities and health promotion campaigns better tailored and personalized to their clients.

#### 4.1.2. Scenario 2: Supporting 2h-OGTT Decision

In this case, the tool would have as input the EHR of a patient, and the main output is to have an estimation of the 2-h OGTT glucose range, given all other available variables. Thanks to this, the tool can support the decision of recommending or not an OGTT, with evident benefits in terms of health outcomes and cost savings.

### 4.2. Technical Assessment

Clinical staff from Hospital La Fe (Table 6) used the system to identify risk sub-groups and to analyze high-low risk subjects during nine consecutive weeks (Table 7).

The technical assessment of the components while running has been evaluated with the deployed version of the system for pilots. The Models Host is running on a Windows Server 2012 R2 Standard, with an Intel${}^{®}$ Xeon${}^{®}$ processor E5405 2 GHz with a RAM memory of 2.35 GB. Performance and resource utilization have been monitored using the Choreographer Logger Service and default Windows/Ubuntu Performance Analysis Tools. A routine for the execution of each model was launched ten times while key performance indicators were recorded. The highest and lowest values have been removed, and the average of the following eight has been calculated and reported in this section.

### 4.3. Map of Evaluations

The system is a service-oriented architecture composed of three main modules: (1) Data Storage Module; (2) Model Host Module; and (3) Interface Module. Several components deployed in different technologies conform to each of these modules, and the collaboration and smooth communication among them were critical issues to guarantee the proper execution of the defined workflows. The evaluations have been done as the study clinical scenarios (Section 4.1), but more specifically, the components affected are:

- Data Warehouses (DW)
- Data Access Layer (Query Engine (QE)): multiple/single subject
- Missing Data Imputation (MDI)
- Risk Score Module (RSM)
- Orchestrator (O)
- Interface Module

The execution of the mentioned components did not follow a subsequent schema, as some of them operate in the background and update new information or model outcomes as they are ready to be sent to related components (for instance, the QE checks if result data are already available from previous requests and displays cached results, without invoking MDI/RSM again).

Technical performance has been done on the mentioned components and looking for the following indicators:

- Verification of the model execution:
  - Appropriateness of the query
  - Units homogenization
  - Handling the resultsand storage
- Performance of the model execution:
  - Best case vs. worst case
  - Latency (time delay of the response)
  - Memory Load in the system server
  - Central Process Unit (CPU) load
  - Network resources

### 4.4. Verification of the Models’ Execution

**Figure 7:**
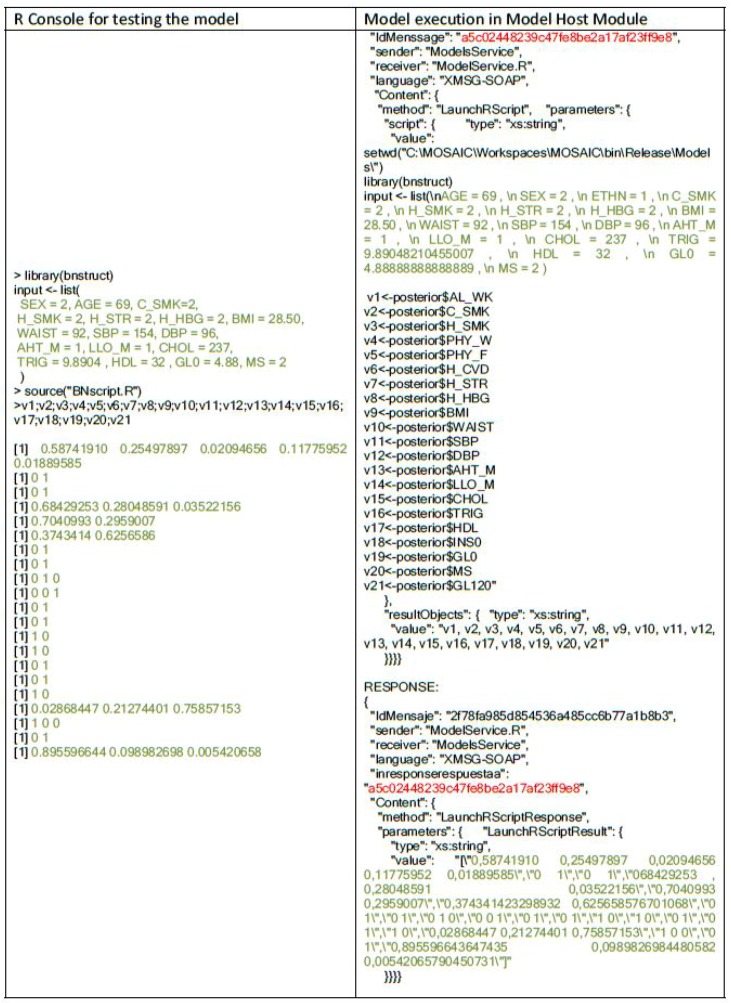
Comparison of the isolated and integrated execution of the Data Imputation R script.

The evaluated system has integrated the state of the art statistical models (risk scores and data imputation) as their own scripts and not as executable files. By this, the system can overtake hot-updates (without stop-reset) and minor modifications easily (re-calibration). Figure 7 shows an example of the missing data imputation model integration: on the left side, the original code; on the right side, the integration script, which implements a call to the R engine and the raw script file. As a matter of integrating raw code, there were some verifications to be done in the way that the risk scores were implemented an, moreover, in the way the variables have to be given as input.

#### Model Integration

The first step is to check that the script (or set of scripts) that was going to execute the statistical engine (R and/or MATLAB) was correctly formatted. To check this, the track service in the Choreographer provides a trace of the messages exchanged among system components and their content. Prior to the system release and in the development version of the system, a query for each of the models is executed, and the trace message is analyzed, as described in Figure 7.

Technical assessment was done for two boundary scenarios (best and worst case depicted in Table 8). Results are provided in tables and figures, which stand for a 60-s time window of the described operations.

In Table 8, the worst case for the data imputation model happens when there are no imputation parameters, so the Bayesian network has to perform all the operations to estimate unknown variables; whereas, if the model has all the input variables (20 for the best case), no estimating operation is needed.

**Figure 8:**
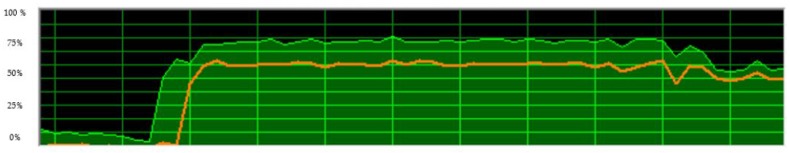
CPU relative use (%) for the prediction risk score execution under the worst case (Orange Line).

**Figure 9:**
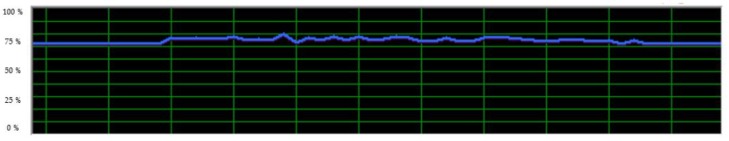
Memory use of the prediction model execution for the worst case. Memory burst occurs due to pagination when the model is executed.

**Figure 10:**
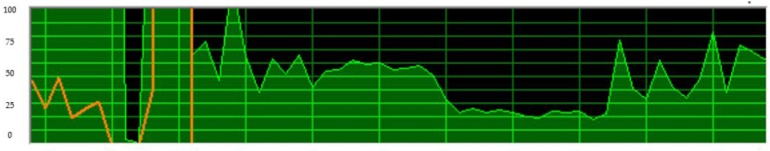
Network resources of the prediction risk score execution for the worst case (orange line). Over-buffering occurs due to the auto-scale mode of the monitor. Peak = 175,296 kpbs.

Figure 8, Figure 9 and Figure 10 show the execution performance of a risk score for the worst case (execution over 8080 subjects). The CPU is used for an average of 60.5% during 25.876 s. No interruptions are produced by memory allocations, network issues or CPU overflow.

Table 9 shows the performance of the database engine for each of the clinical services (data marts in the data warehouse). The CPU average use is 43.70%, and the latency depends on the number of subjects that have to be uploaded. The worst case is found for loading laboratory data for 6402 subjects, which takes 248 min for the setup loading and 3.462 s for subsequent queries. This fact may be originated by the data size and not because of the query engine, which maps the ontology concepts to the specific data attributes in this data mart.

Figure 8 shows the CPU use (%) during the execution of a prediction risk score based on a logistic regression model for the worst case. The orange line represents the Choreographer process, which wraps the model script and executes the algorithm for n = 8080 patients. The CPU usage is under the threshold of 83%, which stands under the target for a proper execution [44].

Figure 9 shows the memory use for the same case. The use of a distributed architecture prevents memory overload, as the Choreographer queues the requests for model execution. However, the figure shows a slight burst that happen because of the automatic memory pagination done by the operative system.

Figure 10 shows at the same time how the network resources are managed. The Choreographer (orange line) performs a query to the Data Storage module to retrieve the data from the n = 8080 patients, which leads to a short period of high data transference (175,296 kpbs). After retrieving the data to execute the models, the module remains without further network demands.

## 5. Discussion

Healthcare systems should shift to perform proactive campaigns on health promotion and disease prevention. The explosion of HIS, data storage technologies and artificial intelligence has paved the way to face the challenges of a progressively aging and sedentary population. However, there is still a gap between research outcomes and clinical applications. In this manuscript, we have presented the results of a pilot study on the implementation of a distributed architecture aimed to integrate artificial intelligence with EHR and to provide decision support tools to clinicians to identify T2DM high risk subjects in real clinical settings. The system used in the study was successful in enabling the use of clinical records to evaluate the performance of seven different state-of-the-art risk scores to detect T2DM high risk subjects. Some minor technical issues were raised at the start of the evaluations; however, thanks to the approach of providing a distributed service-oriented architecture, these could be quickly resolved without affecting the pilot execution, enabling support of the principles of the evidence-based medicine paradigm.

The reasons why clinicians and researchers are not prone to use predictive modeling are identified as a lack of reliability and inadequacy of the models’ validation, as in most cases, this is done just as internal validation. The distributed hybrid architecture proposed herein implements a centralized coordination of services by merging the main three components needed to overcome the aforementioned barriers [50]. By defining a common exchange messaging format and a semantic definition of the services, the proposed architecture is capable of modifying the interaction flow to improve the outcome. Using I2B2 technology, EHR data were integrated by the definition of a common ontology that embraces all the different parameters across them in the Data Storage Module. Next to this module, the Model Host Module gathers the internally-validated discrimination (Bayesian network) and predictive (logistic regression/Cox survival model) models to be assessed with the data sources for the data side. These modules used the model script code to generate automatically the executable model to be used in the discrimination and prediction of T2DM independently. This approach permitted making improvements in the model performance without the need for re-debugging the entire module. In these terms, a model can be externally validated within the same system infrastructure and, thus, be provided to the end users through the integration of the discrimination or predictive tool in the current software management system used in the clinical setting.

Among the several solutions to perform system integration, the one described in this manuscript has the strength of connecting with isolated specific services such as the I2B2 DW, R/MATLAB mathematical engines and web interfaces, allowing one to perform the integration of multiple models towards the early detection of T2DM using hybrid modeling techniques. The applications tested in the pilot study were focused on the execution of risk scores for T2DM; nonetheless, the modular approach driven by the choreographed architecture allowed us the integration of other types of applications, models and databases. We could therefore ensure that hybrid models work together, preventing system faults, exceptions and excessive lag times. This resulted in a smooth workflow of actions that could result in the increased satisfaction of its use by the clinical staff.

The type of models evaluated in this study are based on mathematical and probabilistic equations, which are easy to execute on reduced population sizes, but hard on large populations [51]. The approach of distributing the operations for data storage and model execution was crucial to achieve a reasonable technical performance. Apart from the technical limitations, depending on the data warehouse and mathematical engines needed, the deployment could require the clinical facility to purchase special software licenses for executing the models (R is license free, but MATLAB is not). Moreover, the use of web-clients as graphical user interfaces allowed clinicians to access to the tools from any computer and tablet. Computers in the clinical offices have very limited computational resources, so leveraging them only to interact with a resource-demanding back-end resulted in an efficient execution of ETL operations and risk model evaluation.

The presented architecture implements a message exchange protocol (XMGS), which is based on the FIPA protocol [49], containing meta-data in which services models can be expressed, identified and traced. XMSG is a conceptual framework that has been implemented in XML messages, but it could be converted to JSON and RESTful formats by the integrating converters

A service-oriented architecture provides the framework to dynamically connect distributed services. The service provider and service consumer must rely on and trust each other to successfully complete an operation. In our case, for a screening action, a clinician could consider that executing a risk score she/he knows to be the best performing provides the best predication accuracy. Even though the operation could be successfully completed because of a proper logical connection, it could happen that the selected model is not the one with the highest accuracy or performance. In our study, we have assessed the functional requirements in terms of technical KPI; however, the trustworthiness and quality of the services should be also assessed using dynamic web service selection technologies. This paradigm of dynamic service connection and semantic search may be based on agent-based solutions, in which each agent (former service) contains a semantical description and meta-data on the technical KPIs (such as the C statistic, accuracy, response time, reliability and availability). The implementation of the Web Services Agent Framework (WSAF) [52] incorporating service selection agents that use the QoS ontology would allow assessing and verifying whether each consumer selected the best fitting services, not only in the screening of T2DM, but in the integration of hybrid models in other types of diseases and clinical applications.

The use of semantic information in the service registry would allow one to know the semantic meaning of the input and the type of expected answer that may be returned. This casuistic information can be used by a reasoner to allow the services to use semantically-driven searches to improve search accuracy by understanding the contextual meaning of service terms. Each service would provide information using semantic languages like the Ontology Web Language (OWL) and WSAF, for instance the type of model, the type of input variables and their units and the statistical performance of the model (S and Sp). This information would be used by the reasoner to discover services that match not only the syntactical information, but also the meaning, with the high level query of the user or the service. This type of system would allow retrieving context-based search results that make the system more dynamic and powerful, helping computers to perform automated information gathering and research.

A limitation of the proposed architecture is that it ponders the flexibility over the efficiency. If the risk scores and imputation algorithms were not to require changes, the better solution would have been to integrate an executable file based on C/C++ or interoperability frameworks [53], gaining efficiency and resources use. However, one of the main requirements was to provide a flexible architecture, capable of modifying in the runtime environment the parameters of the models and even the data queries. We implemented therefore the wrapper service, which enabled a dynamic modification of the algorithms and tracing the the data flow with transparency (Figure 7).

Future work will be focused on the expansion of inter-operable services to collect data from personal health records and overcome the hurdles of incomplete EHRs to have a clear picture of the patients’ evolution outside the clinical setting. Moreover, the pilot study should be scaled to a large study including more clinicians from different units testing more predictive algorithms.

## 6. Conclusions

The present manuscript has described the usefulness and reliability of a choreographed service architecture to integrate hybrid models with clinical settings. Evidence-based medicine requires technological frameworks to implement and test research outcomes within clinical scenarios. T2DM risk models have been shown to perform well on retrospective and prospective clinical trials; however, their performance is still unknown using population datasets. Our pilot study has confirmed the capability of a distributed system based on service choreography to integrate heterogeneous modeling techniques, clinical data sources and web-based user interfaces, which paves the way toward the implementation of evaluation studies based on real population datasets.

## Figures and Tables

**Table 1:** System reference success criteria.

RSC ID	RSC Description
RSC#1	Supporting rich human computer interaction
RSC#2	Supporting intelligent hardware abstraction
RSC#3	Enabling system-driven interaction
RSC#4	Supporting continuity of care
RSC#5	Supporting end user security and privacy
RSC#6	Supporting the update, set up and management of system components
RSC#7	Supporting remote/local operation
RSC#8	Supporting data granted access to perform CRUD (Create, Replace, Update, Delete) operations
RSC#9	Interface existing information systems
RSC#10	Supporting service providers to offer system services
RSC#11	Allowing users to find system tools
RSC#12	Supporting exploitation of different business models
RSC#13	Capturing and utilizing user feedback
RSC#14	Supporting rapid development of new models
RSC#15	Model-based development of services through integrated model transformation tools
RSC#16	Supporting on-line elicitation of requirements and the collection of runtime feedback from users of risk score services
RSC#17	Supporting advanced search, reuse and sharing of service components and resources
RSC#18	Supporting customization of system services

**Table 2:** Types of service collaboration patterns among system components.

Types of Services	Description
Module-to-Module (B2B)	Services that are provided by one system module to other module(s) of a different type (e.g., a web service provider from the models requires data from a remote database service provider).
Module-to-Client (B2C)	Services that are provided by a module to client stakeholders (e.g., a web service provider provides remote execution of a model).

**Table 3:** Comparison of state of the art solutions for data storage and the feature each engine provides for performing queries. Among the compared engines, Informatics for Integrating Biology and the Bedside (I2B2) does not support unstructured storage, but is the one capable of abstracting the concepts into an ontology.

	Open Source	Structured Storage	Unstructured Storage	Scalability	Ontologies
MongoDB	YES	NO	YES (Json)	YES	NO
Hadoop	YES	YES	YES	YES	NO
OracleDB	NO	YES	YES -for Oracle NoSQL	YES	NO
MySQL	YES	YES	NO	Compromised	NO
SQLServer	NO	YES	NO	Compromised	NO
I2B2	YES	YES	NO	Compromised	YES
Cassandra	YES	NO	YES	YES	NO

**Table 4:** Discrimination performance of state of the art risk scores to be assessed. FG: Fasting Glucose; AHT: Anti-HyperTensivemedication; HDL: High-Density Lipoprotein; FHD: Family History of Diabetes; BMI: Body Mass Index; LL: Lipid-Lowering medication.

Risk Score Name and Validation Study	Mathematical Model	Performance (*C statistic*)	Predictors
Findrisc [32,33]	Weighted Logistic Regression	85%	Age, AHT medication, FG, BMI, Waist
ARIC [20,34]	Logistic Regression	80%	Age, Ethnicity, FG, HDL, Triglyceride, Blood Pressure, FHD, Waist, Height
San Antonio [35,36]	Linear Regression	84%	Age, Gender, Ethnicity, FG, BMI, HDL, Blood Pressure, FHD
Cambridge [21,37]	Logistic Regression	75%	Age, Gender, AHT, Steroids, BMI, FHD, Smoking habit
PREDIMED [38]	Multivariate Cox Survival Model	78%	AHT, FG, Blood Pressure, FHD, Smoker, Alcohol Intake
Framingham [34,39]	Logistic Regression	84%	Age, Gender, AHT, FG, BMI, HDL, Triglyceride, Blood Pressure, FHD, Waist
MOSAIC [40]	Bayesian Network	79%	Age, Gender, FG, Smoker, Alcohol, AHT, LL, Physical Activity, Triglyceride, HDL, BMI, Waist, Stroke, FHD

**Table 5:** XMSG example.

REQUEST:,20/09/2017 13:03:55.802 {“IdMessage”: “b8df1baf178043539cad47beea3a51e2”, “sender”: “ModelsService”, “receiver”: “ModelService.R”, “Credential”: token, “language”: “XMSG-SOAP”, “inresponse”, “Content”: { “method”: “LaunchRScript”, “parameters”: { “script”: { “type”: “xs:string”, “value”: “setwd”(“C:\CHOREOGRAPHER\WorkingDirectoryR”), v0=c(SubjID = 21, SEX = 2, AGE = 76, ETHNIC = 1, WAIST = 100, PULSE = 80, DBP = 70,,HOMA_B = 0, HOMA_IR = 0, GLUC0 = 5, TRIG = 1.5909090, MS = 2, PHYSICAL_WORK = 1,CURR_SMOKE = 2, MAR_MARR = 1, MAR_DIV = 2, MAR_WID = 2, PROF_NONE = 2), v0df=as.data.frame(t(v0)), v1=c(SubjID = 22, SEX = 1, AGE = 54, ETHNIC = 1, WAIST = 111, PULSE = 80, DBP = 80,,HOMA_B = 0, HOMA_IR = 0, GLUC0 = 7.222222, TRIG = 1.05681, MS = 1, PHYSICAL_WORK = 1,CURR_SMOKE = 2, MAR_MARR = 2, MAR_DIV = 2, MAR_WID = 1, PROF_NONE = 2), v1df=as.data.frame(t(v1))}, “resultObjects”: {“type”: “xs:string”, “value”: “pd1, pd2”}}}}
RESPONSE:,20/09/2017 13:03:55.834 {“IdMessage”: “e624f00474074b5fad810c014ff4a62e”, “sender”: “ModelService.R”, “receiver”: “ModelsService”, “Credential”: token, “language”: “XMSG-SOAP”, “inresponse”: “b8df1baf178043539cad47beea3a51e2”, “Content”: {,“method”: “LaunchRScriptResponse”, “parameters”: “LaunchRScriptResult”: {“type”: “xs:string”, “value”: “[0,000212 0,000446 0,000731 0,001271 0,002155 0,004113 0,0054412 0,0064554 0,008481 0,0122504 0,018581; 0,001776 0,0037354 0,00611205 0,010603 0,017916 0,0339384 0,044676 0,052806 0,068869 0,098103 0,1453966}}}}

**Table 6:** Clinicians included in the pilot study to evaluate the two scenarios.

**Gender**		Male (2)/Female (6)
**Age (Years)**		42 ± 13
**Professional Experience (Years)**		14 ± 10
**ICT Literacy (Self-reported)**		High = 3; Medium = 3; Low = 2;
**Number of Patients Assisted**	Overall	319.33 ± 247.66
	T2DM Patients	127.44 ± 75.22
	High risk of developing T2DM	48.00 ± 33.79

**Table 7:** Distribution of the evaluation sessions (number, duration, number of patients per day and per session).

Indicator of Use	*Mean*	*SD*	*Min*	*Max*
Number of users per day	2.5	16.43	1	4
Duration of sessions (min)	26.16	13.72	0.25	45.93
Number of patients evaluated per doctor	6.25	4.97	1	15
Number of patients evaluated per day	10.71	12.18	0	26
Number of sessions per doctor (user)	1.82	1.16	1	5

**Table 8:** Results of the technical assessment for the best and worst scenario in the prediction risk score and the data imputation model.

**Prediction Risk Score**					
	**n**	**Latency (s)**	**CPU(%)**	**Memory (kB)**	**Bandwith (kbps)**
**Best Case**	1	0.016	20.20	374,012	9.8
**Worst Case**	8080	25.876	60.50	463,853	173.35
**Data Imputation Model**					
	**Input Vars**	**Latency (s)**	**CPU(%)**	**Memory (kB)**	**Bandwith (kbps)**
**Best Case**	20	1.486	48.50	360,416	40.23
**Worst Case**	0	1.860	49.5	360,748	63.56

**Table 9:** Performance for the Database Management Module among different services and regular queries.

Database Module Performance						
Service	Number of Subjects	Time to Setup (min)	Latency per Patient (s)	CPU (%)	Memory (kb)	Bandwidth (kbps)
Emergency	658	79	7.412	43.70	137,733	720
Outpatient	1020	67	1.766			
Laboratory	6402	248	3.462			
Regular Queries	-	-	0.254	60.20	80,457	72,459

## Abbreviations

The following abbreviations are used in this manuscript:

BN	Bayesian Network
EHR	Electronic Health Record
DW	Data Warehouse
HbA1C	Glycated Hemoglobin
HIS	Hospital Information System
KPI	Key Performance Indicator
T2DM	Type 2 Diabetes Mellitus
SOAP	Simple Object Access Protocol
RSC	Reference Success Criteria
UML	Unified Modeling Language
XMSG	X-Message
2h-OGTT	2 h Oral Glucose Tolerance Test
